# PLGA Based Drug Carrier and Pharmaceutical Applications: The Most Recent Advances

**DOI:** 10.3390/pharmaceutics12090903

**Published:** 2020-09-22

**Authors:** Joana Angélica Loureiro, Maria Carmo Pereira

**Affiliations:** LEPABE, Department of Chemical Engineering, Faculty of Engineering of the University of Porto, s/n, R. Dr. Roberto Frias, 4200-465 Porto, Portugal

Poly(lactic-*co*-glycolic acid) (PLGA) is one of the most successful polymers that has been used to produce medicines, such as drug carriers (DC). This is one of the few polymers that the Food and Drug Administration (FDA) has approved for human administration due to its biocompatibility and biodegradability [1]. DCs produced with PLGA have gained enormous attention over recent years for their ability to be versatile vehicles to transport different type of drugs, e.g., hydrophilic or hydrophobic small molecules or macromolecules, and protect them from degradation and uncontrolled release [2,3,4,5,6]. These drug delivery systems (DDS), including micro and nanoparticles, have the potential to modify their surface properties and improve interactions with biological materials. Furthermore, they can also be conjugated with specific target molecules to reach specific tissues or cells [7,8]. They are being used for different therapeutic applications, such as vaccinations or as treatments for cancer, neurological disorders, inflammation, and other diseases [9,10,11,12].

This Special Issue aims to focus on the recent progress of PLGA as a drug carrier and its new pharmaceutical applications. It comprises an exciting series of 19 research articles on the recent advances in the field.

In the first research study presented in this Special Issue, Ho et al. developed polymeric microspheres which contain micronized triamcinolone acetonide (TA) in order to increase the drug retention time in joints after intra-articular administration [13]. Poly(lactic-*co*-glycolic acid)/poly(lactic acid) (PLGA/PLA) carriers were prepared through spray-drying to incorporate the microcrystals that were previously prepared by ultra-sonication. In vivo testing in rat models was demonstrated to prolong drug retention in joints. The TA remained there for over 28 days, which was more 21 days compared with the TA-free group. Furthermore, these nanocarriers were demonstrated to be stable for one year.

The group of Peula-García used PLGA nanoparticles to carry bone morphogenetic protein (BMP-2) [14]. The nanocarriers were synthetized by a double-emulsion (water/oil/water, W/O/W) solvent evaporation technique, using the surfactant Pluronic F68 as a stabilizer. The BMP2-loaded nanocarriers presented positive results when evaluated using mesenchymal stromal cells from human alveolar bone regarding their proliferation, migration, and osteogenic differentiation. Another strategy to encapsulate BMP-2 was conducted by Minardi et al. [15]. PLGA multistage vector composite microspheres were used as carriers that demonstrated a good capacity for BMP-2 encapsulation and did not present toxicity for the rat mesenchymal stem cells. García-García et al. applied a combined strategy to regenerate tissue defects [16]. They used BMP-2- and 17β-estradiol-loaded microspheres, PLGA-based, in a sandwich-like system produced by a hydrogel core.

In another study, Hwang et al. fabricated PLGA carriers combined also with a hydrogel matrix. They produced oxaliplatin-loaded PLGA microparticles using a double emulsion technique and then loaded them into hyaluronic acid and carboxymethyl cellulose sodium-based cross-linked hydrogels [17]. This drug delivery system was analyzed in rat models and a substantial improvement was observed in terms of bioavailability and the mean residence time of the microparticle-loaded hydrogels.

Kim et al. developed an original system to be used in the topical delivery of trolamine salicylate (TS), a topical anti-inflammatory analgesic used for the treatment of small joint pain [18]. Here, the optimization process was done using different amounts of PLGA, ethyl 2-cyanoacrylate, poly (ethylene glycol) (PEG) 400, and TS. The researchers proved that the produced formulations enhanced the delivery of TS into and across the skin.

Duse et al. used PLGA nanoparticles to encapsulate curcumin, a well know natural compound that present anticancer benefits [19]. It was shown that the use of PLGA nanoparticles improves the bioavailability and site-specific drug uptake. The nanoparticles’ efficacy was tested using SK-OV-3 human ovarian adenocarcinoma cells and demonstrated to be very efficient in transporting curcumin. Furthermore, with the same objective to treat the cancer, our research group used factorial design as a tool to optimize the co-encapsulation of temozolomide and o6-benzylguanine in PLGA nanoparticles [20]. The produced nanoparticles, rather than demonstrating stability for several days, presented optimal physicochemical properties for brain delivery, including a size lower than 200 nm and a negative surface charge. In the same research line, demonstrating the potential of the co-encapsulation, Bazylińska et al. encapsulated a hydrophobic porphyrin photosensitizing dye—verteporfin—in combination with low-dose cisplatin, a hydrophilic cytostatic drug [21]. Different coatings were applied to the PLGA nanoparticles, PEG, or folic acid functionalized. Those nanoparticles proved to have an increased internalization and efficiency regarding anticancer activity.

Another interesting study proposed by Varga and colleagues, who contributed with an interesting study of nanoparticle design and optimization where the (±)-α-Tocopherol (TP) with vitamin E activity was encapsulated in PLA and PLGA nanoparticles [22]. To stabilize the nanoparticles, the non-ionic stabilizing surfactant Pluronic F127 was used. Several techniques were used to characterize these novel nanoparticles, such as transmission electron microscopy (TEM), dynamic light scattering (DLS), and infrared spectroscopy (FT-IR).

Morelli et al. improved paclitaxel delivery in the gastro-intestinal tract by encapsulating the drug in PLGA nanoparticles coated with PEG [23]. The nanoparticles demonstrated stability in the gastric tract and simply penetrated inside carcinoma colon 2 (CaCo_2_) cells.

With the objective to overcome the undesired lag time of the commercially available risperidone, Janich et al. encapsulated this drug in PLGA–lipid microcapsules and PLGA–lipid microgels [24]. The carriers were evaluated regarding their physio-chemical properties and the first formulation was demonstrated to achieve a nearly zero order release without a lag time over 2 months.

A research work using PLGA nanoparticles for ocular application was also collected. Ryu et al. produced rapidly dissolving dry tablets containing alginate and dexamethasone-loaded PLGA nanoparticles [25]. These nanoparticles presented sustained drug release for 10 h. In vivo experiments showed their efficiency and make this DDS a promising strategy for aseptic and accurate dose delivery of ophthalmic drugs.

An interesting approach based on a combination of cell and drug delivery for the treatment of Huntington’s disease (HD) was proposed by André et al. [26]. The authors used laminin-coated PLGA nanoparticles to transport brain-derived neurotrophic factor (BDNF). The nanoparticles/cell complexes were evaluated in an ex vivo model of HD. Promising results were obtained by the researchers, encouraging them to go further in their research with this system.

Two works lead by Roing and Wacker present new theranostic PLGA-based nanoparticles. In the first one, biodegradable and photoluminescent polyester (BPLP) with PLGA polymer was used to fabricate biocompatible photoluminescent nanocapsules [27]. Additionally, superparamagnetic iron oxide nanoparticles (SPIONs) were incorporated into the polymeric shell to transform the particles into a magnetic resonance/photoluminescence dual-model imaging theranostic platform. The particles demonstrated good uptake and biocompatibility with hCMEC/D3 endothelial cells. In the second study, three different technologies for the encapsulation of sorafenib into PLGA and PLGA–PEG copolymers were adopted [28]. Those nanoparticles presented size ranges between 220 and 240 nm. In order to transform those nanoparticles in a theranostic medicine, gadolinium complexes were covalently attached to the nanoparticles’ surface. That way, the nanoparticles could be located using magnetic resonance imaging.

PLGA toxicity was investigated by Bakhaidar et al. [29]. Here, the researchers studied the impact of size-selected PLGA–PEG nanoparticles on platelet activation and aggregation. The results demonstrated that nanoparticles of all sizes are associated with the surface of platelets leading to possible internalization. Furthermore, the NP–platelet interaction proved to not conduct platelet aggregation, making these PLGA nanoparticles promising delivery systems for targeted drug delivery to platelets.

Another relevant study was performed by Operti et al., who used microfluidics technology as a tool to manufacture particles in a highly controllable way [30]. In their study, they produced PLGA particles at diameters ranging from sub-micron to micron using a single microfluidics device. Through modification of flow and formulation parameters, the nanoparticle size changed substantially. Furthermore, in this study, the researchers proved how the particle size influences the release characteristics, cellular uptake, and in vivo clearance of these particles.

Finally, a research study regarding the importance of new techniques to characterize PLGA nanoparticles was included in this special edition. Shmool et al. investigated the dynamics of PLGA microspheres prepared by freeze-drying [31]. The water-oil-water (w/o/w) double-emulsion technique was selected for the production of the microspheres. Their molecular mobility at lower temperatures, leading to the glass transition temperature, using temperature-variable terahertz time-domain spectroscopy (THz-TDS), was evaluated. THz-TDS records show distinct transition processes, one in the range of 167–219 K, associated with local motions, and the other in the range of 313–330 K, associated with large-scale motions.

The papers presented in this Special Issue represent a small part of the research that is ongoing in the field of PLGA nanocarriers all over the world. The huge potential of PLGA nanoparticles make them a promising drug delivery system with outstanding properties and with much more potential for exploring in the coming years. With this Special Issue, the editors expect that the readers from the field find it stimulating and contributing more ideas or methodologies for their future work.

## References

[1] Makadia H.K., Siegel S.J. (2011). Poly Lactic-*co*-Glycolic Acid (PLGA) as Biodegradable Controlled Drug Delivery Carrier. Polymers.

[2] Ramalho M.J., Sevin E., Gosselet F., Lima J., Coelho M., Loureiro J.A., Pereira M. (2018). Receptor-mediated PLGA nanoparticles for glioblastoma multiforme treatment. Int. J. Pharm..

[3] Olivier J.-C. (2005). Drug Transport to Brain with Targeted Nanoparticles. NeuroRX.

[4] Rezvantalab S., Drude N.I., Moraveji M.K., Güvener N., Koons E.K., Shi Y., Lammers T., Kiessling F. (2018). PLGA-Based Nanoparticles in Cancer Treatment. Front. Pharmacol..

[5] Ramalho M.J., Loureiro J.A., Gomes B., Frasco M.F., Coelho M.A.N., Pereira M.D.C. (2015). PLGA nanoparticles as a platform for vitamin D-based cancer therapy. Beilstein J. Nanotechnol..

[6] Lu B., Lv X., Le Y. (2019). Chitosan-Modified PLGA Nanoparticles for Control-Released Drug Delivery. Polymers.

[7] Jose S., Cinu T.A., Sebastian R., Shoja M.H., Aleykutty N.A., Durazzo A., Lucarini M., Santini A., Souto E.B. (2019). Transferrin-Conjugated Docetaxel–PLGA Nanoparticles for Tumor Targeting: Influence on MCF-7 Cell Cycle. Polymers.

[8] Loureiro J.A., Gomes B., Fricker G., Coelho M.A.N., Rocha S., Pereira M.D.C. (2016). Cellular uptake of PLGA nanoparticles targeted with anti-amyloid and anti-transferrin receptor antibodies for Alzheimer’s disease treatment. Colloids Surf. B Biointerfaces.

[9] Guarecuco R., Lu J., McHugh K.J., Norman J.J., Thapa L.S., Lydon E., Langer R., Jaklenec A. (2018). Immunogenicity of pulsatile-release PLGA microspheres for single-injection vaccination. Vaccine.

[10] Shen X., Li T., Xie X., Feng Y., Chen Z., Yang H., Wu C., Deng S., Liu Y. (2020). PLGA-Based Drug Delivery Systems for Remotely Triggered Cancer Therapeutic and Diagnostic Applications. Front. Bioeng. Biotechnol..

[11] Rigon L., Salvalaio M., Pederzoli F., Legnini E., Duskey J.T., D’Avanzo F., De Filippis C., Ruozi B., Marin O., Vandelli M.A. (2019). Targeting Brain Disease in MPSII: Preclinical Evaluation of IDS-Loaded PLGA Nanoparticles. Int. J. Mol. Sci..

[12] Deng M., Tan J., Hu C., Hou T., Peng W., Liu J., Yu B., Dai Q., Zhou J., Yang Y. (2020). Modification of PLGA Scaffold by MSC-Derived Extracellular Matrix Combats Macrophage Inflammation to Initiate Bone Regeneration via TGF- β -Induced Protein. Adv. Heal. Mater..

[13] Ho M.J., Jeong H.T., Im S.H., Kim H.T., Lee J.E., Park J.S., Cho H.R., Kim D.Y., Choi Y.W., Lee J. (2019). Design and In Vivo Pharmacokinetic Evaluation of Triamcinolone Acetonide Microcrystals-Loaded PLGA Microsphere for Increased Drug Retention in Knees after Intra-Articular Injection. Pharmaceutics.

[14] Del Castillo T., Ortega-Oller I., Padial-Molina M., O’Valle F., Galindo-Moreno P., Jodar-Reyes A.B., Peula J. (2019). Formulation, Colloidal Characterization, and In Vitro Biological Effect of BMP-2 Loaded PLGA Nanoparticles for Bone Regeneration. Pharmaceutics.

[15] Minardi S., Fernandez-Moure J.S., Fan D., Murphy M.B., Yazdi I.K., Liu X., Weiner B.K., Tasciotti E. (2020). Biocompatible PLGA-Mesoporous Silicon Microspheres for the Controlled Release of BMP-2 for Bone Augmentation. Pharmaceutics.

[16] García-García P., Reyes R., Segredo-Morales E., Herrero E., Delgado A., Évora C. (2019). PLGA-BMP-2 and PLA-17β-Estradiol Microspheres Reinforcing a Composite Hydrogel for Bone Regeneration in Osteoporosis. Pharmaceutics.

[17] Abuzar S.M., Ahn J.-H., Park K.S., Park E., Baik S., Hwang S.-J. (2019). Pharmacokinetic Profile and Anti-Adhesive Effect of Oxaliplatin-PLGA Microparticle-Loaded Hydrogels in Rats for Colorectal Cancer Treatment. Pharmaceutics.

[18] Kim Y., Beck-Broichsitter M., Banga A.K. (2019). Design and Evaluation of a Poly(Lactide-*co*-Glycolide)-Based In Situ Film-Forming System for Topical Delivery of Trolamine Salicylate. Pharmaceutics.

[19] Duse L., Agel M.R., Pinnapireddy S.R., Schäfer J., Selo M.A., Ehrhardt C., Bakowsky U. (2019). Photodynamic Therapy of Ovarian Carcinoma Cells with Curcumin-Loaded Biodegradable Polymeric Nanoparticles. Pharmaceutics.

[20] Ramalho M.J., Loureiro J.A., Coelho M.A.N., Pereira M.D.C. (2019). Factorial Design as a Tool for the Optimization of PLGA Nanoparticles for the Co-Delivery of Temozolomide and O6-Benzylguanine. Pharmaceutics.

[21] Bazylinska U., Kulbacka J., Chodaczek G. (2019). Nanoemulsion Structural Design in Co-Encapsulation of Hybrid Multifunctional Agents: Influence of the Smart PLGA Polymers on the Nanosystem-Enhanced Delivery and Electro-Photodynamic Treatment. Pharmaceutics.

[22] Varga N., Turcsányi Á., Hornok V., Csapó E. (2019). Vitamin E-Loaded PLA- and PLGA-Based Core-Shell Nanoparticles: Synthesis, Structure Optimization and Controlled Drug Release. Pharmaceutics.

[23] Morelli L., Gimondi S.R., Sevieri M., Salvioni L., Guizzetti M., Colzani B., Palugan L., Foppoli A., Talamini L., Morosi L. (2019). Monitoring the Fate of Orally Administered PLGA Nanoformulation for Local Delivery of Therapeutic Drugs. Pharmaceutics.

[24] Janich C., Friedmann A., Silva J.M.D.S.E., De Oliveira C.S., De Souza L.E., Rujescu D., Hildebrandt C., Beck-Broichsitter M., Schmelzer C.E., Mäder K. (2019). Risperidone-Loaded PLGA–Lipid Particles with Improved Release Kinetics: Manufacturing and Detailed Characterization by Electron Microscopy and Nano-CT. Pharmaceutics.

[25] Ryu W.M., Kim S.-N., Min C.H., Bin Choy Y. (2019). Dry Tablet Formulation of PLGA Nanoparticles with a Preocular Applicator for Topical Drug Delivery to the Eye. Pharmaceutics.

[26] André E.M., Delcroix G.J., Kandalam S., Sindji L., Montero-Menei C.N. (2019). A Combinatorial Cell and Drug Delivery Strategy for Huntington’s Disease Using Pharmacologically Active Microcarriers and RNAi Neuronally-Committed Mesenchymal Stromal Cells. Pharmaceutics.

[27] Zhang Y., García-Gabilondo M., Rosell A., Roig A. (2019). MRI/Photoluminescence Dual-Modal Imaging Magnetic PLGA Nanocapsules for Theranostics. Pharmaceutics.

[28] Feczkó T., Piiper A., Pleli T., Schmithals C., Denk D., Hehlgans S., Rödel F., Vogl T.J., Wacker M.G., Denk D. (2019). Theranostic Sorafenib-Loaded Polymeric Nanocarriers Manufactured by Enhanced Gadolinium Conjugation Techniques. Pharmaceutics.

[29] Bakhaidar R., Green J., Alfahad K., Samanani S., Moollan N., O’Neill S., Ramtoola Z., Neill O. (2019). Effect of Size and Concentration of PLGA-PEG Nanoparticles on Activation and Aggregation of Washed Human Platelets. Pharmaceutics.

[30] Operti M.C., Dölen Y., Keulen J., Van Dinther E.A.W., Figdor C.G., Tagit O. (2019). Microfluidics-Assisted Size Tuning and Biological Evaluation of PLGA Particles. Pharmaceutics.

[31] Shmool T., Hooper P.J., Schierle G.S.K., Van Der Walle C.F., Zeitler J.A. (2019). Terahertz Spectroscopy: An Investigation of the Structural Dynamics of Freeze-Dried Poly Lactic-*co*-glycolic Acid Microspheres. Pharmaceutics.

